# Predictive value of interim FDG-PET/CT findings in patients with diffuse large B-cell lymphoma treated with R-CHOP

**DOI:** 10.18632/oncotarget.27103

**Published:** 2019-09-10

**Authors:** Kazuhiro Kitajima, Masaya Okada, Kyoko Yoshihara, Tazuko Tokugawa, Akihiro Sawada, Satoshi Yoshihara, Hiroya Tamaki, Yoshihiro Fujimori, Syuji Ueda, Hiroyuki Kawamoto, Junichi Taniguchi, Koichiro Yamakado

**Affiliations:** ^1^ Division of Nuclear Medicine and PET Center, Department of Radiology, Hyogo College of Medicine, Nishinomiya, Hyogo, Japan; ^2^ Division of Hematology, Departments of Internal Medicine, Transfusion Medicine and Cellular Therapy, Hyogo College of Medicine, Nishinomiya, Hyogo, Japan; ^3^ Department of Hematology, Hyogo Prefectural Nishinomiya Hospital, Nishinomiya, Hyogo, Japan; ^4^ Department of Hematology, Uegahara Hospital, Nishinomiya, Hyogo, Japan; ^5^ Department of Radiology, Hyogo College of Medicine, Nishinomiya, Hyogo, Japan

**Keywords:** non-Hodgkin lymphoma, PET-CT, progression-free survival

## Abstract

**Objectives:** To examine the prognostic value of interim 18F-fluorodeoxyglucose positron emission tomography/computed tomography (FDG-PET/CT) findings after 2–4 cycles of rituximab, plus cyclophosphamide, doxorubicin, vincristine, and prednisone (R-CHOP) in patients with diffuse large B-cell lymphoma (DLBCL) receiving standardized treatment.

**Results:** After a median 3.36 years (range 0.33 to 9.14 years), 24 of the 80 patients had documented relapse. In Interim-PET findings, 2-year PFS was significantly shorter for PET-positive as compared with PET-negative patients (50.0% vs. 86.4%; *p* = 0.0012). In End-PET findings, 2-year PFS was significantly shorter for PET-positive as compared with PET-negative patients (25.0% vs. 84.7%; *p* < 0.0001). The positive predictive value (PPV) and negative predictive value (NPV) of Interim-PET for predicting relapse or disease progression were 57.1% and 75.8%, respectively, while those for End-PET were 75.0% and 75.0%, respectively.

**Methods:** Eighty DLBCL patients treated with first-line 6–8 R-CHOP courses regardless of interim imaging findings were enrolled. Each underwent FDG-PET/CT scanning at staging, and again during (Interim-PET) and at the end of (End-PET) therapy. PET positivity or negativity at Interim-PET and End-PET as related to progression-free survival (PFS) was examined using Kaplan–Meier analysis.

**Conclusion:** Mid-treatment FDG-PET/CT findings may be useful for determining disease status in patients with DLBCL undergoing induction R-CHOP chemotherapy, though are not recommended for treatment decisions as part of routine clinical practice.

## INTRODUCTION

Diffuse large B-cell lymphoma (DLBCL), the most common type of non-Hodgkin lymphoma worldwide, has been reported to be curable in about 60–70% of patients treated with standard rituximab, plus cyclophosphamide, doxorubicin, vincristine, and prednisone (R-CHOP) chemotherapy [1]. On the other hand, current salvage therapy strategies seem to be inadequate for nonresponding patients, with only 30% to 35% of resistant or relapsed patients in this rituximab era able to achieve prolonged progression-free survival (PFS) with high-dose chemotherapy followed by autologous stem cell transplantation (ASCT). Thus, a possible good alternative approach might be first-line risk-tailored therapy in patients with poor prognosis.

^18^F-fluorodeoxyglucose positron emission tomography/computed tomography (FDG-PET/CT) is a widely used imaging modality, and known to be reliable for staging and response assessment of aggressive malignant lymphomas, including DLBCL. For assessment of response, FDG-PET adds valuable information to that obtained with CT, because, unlike that modality, FDG-PET findings can often differentiate a viable tumor from posttreatment fibrosis or necrosis. Currently, FDG-PET/CT is the standard recommended method for response assessment at the end of first-line treatment, with FDG positivity at that point considered predictive of survival in patients with malignant lymphoma [2, 3]. Although previous studies have found that application of FDG-PET during therapy (Interim-PET) allows for successful PET-guided management of patients with Hodgkin lymphoma [4, 5], the use of Interim-PET for early response assessment and management of patients with DLBCL remains controversial, with varying results reported [6–20]. In the present study, we examined the predictive value of Interim-PET regarding PFS of DLBCL patients using visual dichotomous analysis.

## RESULTS

This study included 80 DLBCL patients (45 males, 35 females; mean age 64.3 years, range 22–84 years), of whom 38 were stage I or II and 42 were stage III or IV. After a median 3.36 years (0.33 to 9.14 years), 24 were documented as relapse cases. Of the 24 patients with recurrence or progression, the follow-up period was a median 1.74 years (0.33 to 5.74 years), while that was a median 3.73 years (1.84 to 9.14 years) for the 56 without recurrence or progression.

**Figure 1:**
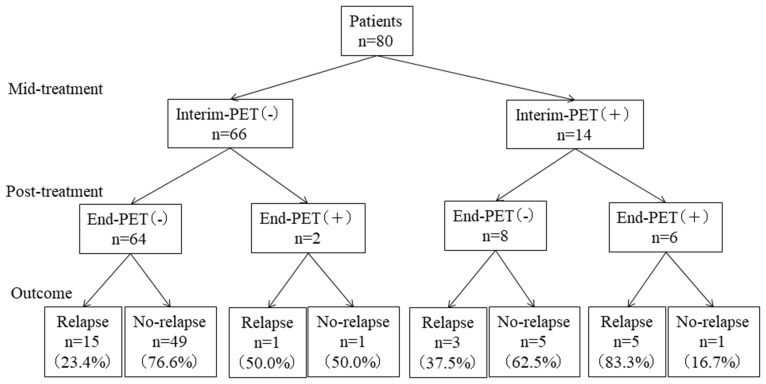
Patient-outcomes according to Interim-PET and End-PET.

An Interim-PET score of 1 was noted in 41 patients, a score of 2 was noted in 11, a score of 3 was noted in 14, a score of 4 was noted in 10, and a score of 5 was noted in 4. As for End-PET, a score of 1 was noted in 49 patients, a score of 2 in 17, a score of 3 in 6, a score of 4 in 6, and a score of 5 in 2. Therefore, 66 patients (82.5%) were negative and 14 (17.5%) were positive at the time of Interim-PET, and 72 (90.0%) were negative and 8 (10%) were positive at the time of End-PET. Eight of 14 (57.1%) patients positive in Interim-PET findings were negative in End-PET results, whereas only 2 of 66 (3.0%) Interim-PET negative cases were positive at End-PET (Figure 1).

**Figure 2:**
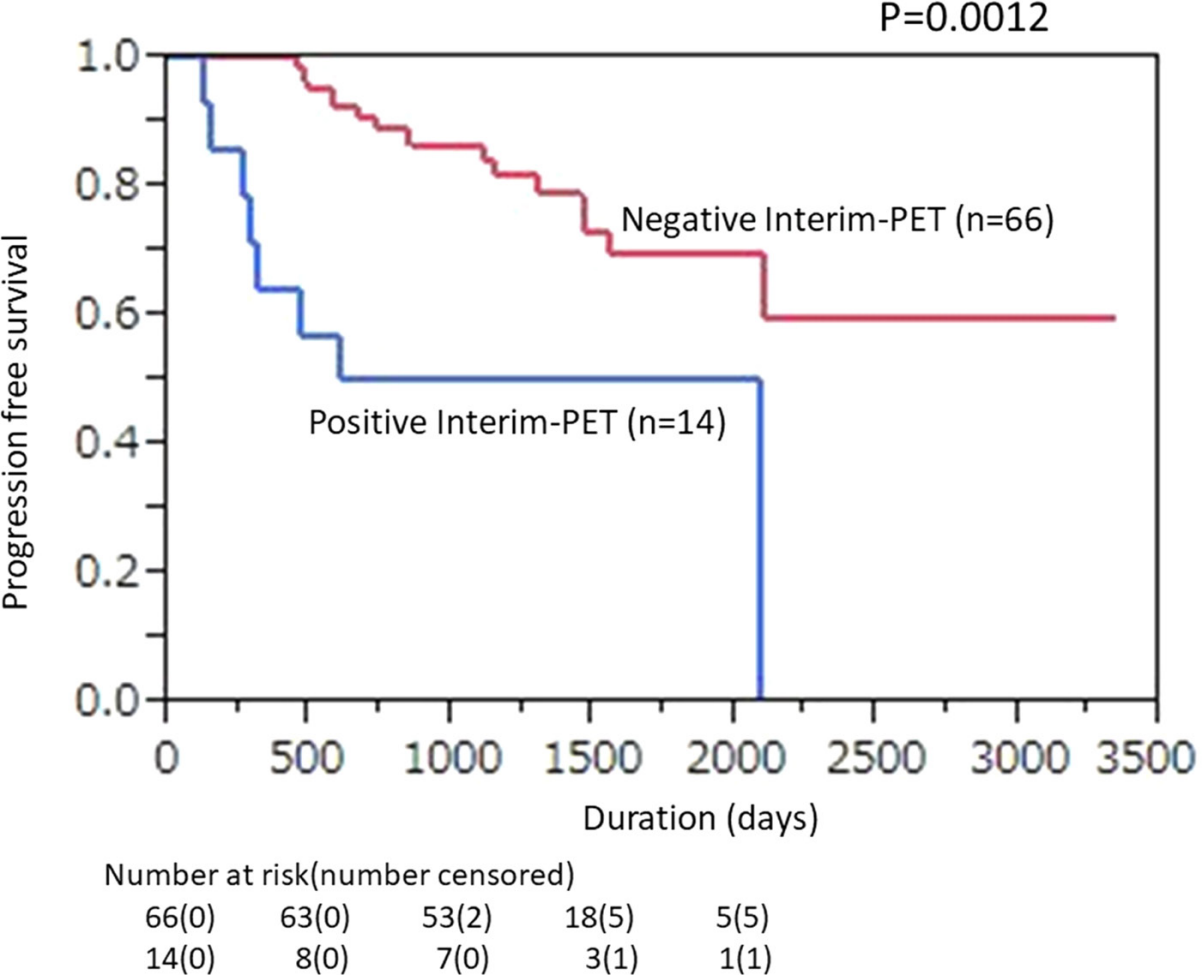
Kaplan–Meier plot showing progression-free survival (PFS) according to mid-therapy FDG-PET/CT findings in patients with diffuse large B-cell lymphoma (DLBCL) treated with 6–8 courses of rituximab, plus cyclophosphamide, doxorubicin, vincristine, and prednisone (R-CHOP). Correlations of Interim-PET results (positivity vs. negativity) with PFS are shown (*p =* 0.0012).

**Figure 3:**
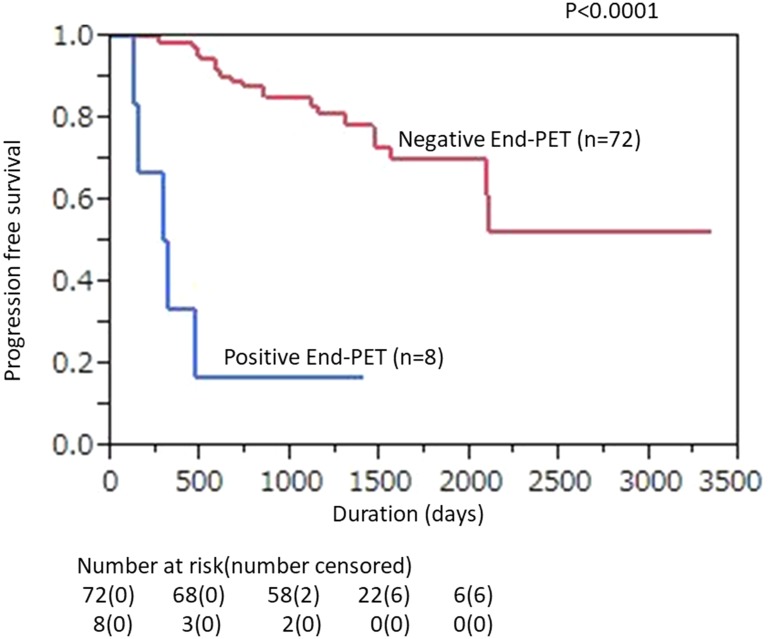
Kaplan–Meier plot showing PFS according to posttherapy FDG-PET/CT findings in DLBCL patients treated with 6-8 R-CHOP courses. Correlations of End-PET results (positivity vs. negativity) with PFS are shown (*p* < 0.0001).

The ratio of 2-year PFS shown by Interim-PET/CT findings was significantly lower for PET-positive as compared with -negative patients (50.0% [95% CI, 23.8% to 76.2%] *vs.* 86.4% [95% CI, 78.1% to 94.6%]; *p =* 0.0012, Figure 2). In End-PET/CT findings, 2-year PFS was also significantly lower for PET-positive as compared with -negative patients [25.0% (95% CI, 0% to 55.0%) *vs.* 84.7% (95% CI, 76.4% to 93.0%); *p <* 0.0001, Figure 3].

The rates for PPV, NPV, sensitivity, and specificity for Interim-PET to predict relapse or progression were 57.1% (95% CI, 31.2% to 82.9%), 75.8% (95% CI, 65.4% to 86.1%), 33.3% (95% CI, 14.5% to 52.2%), and 89.3% (95% CI, 81.2% to 97.4%), respectively, while those for End-PET were 75.0% (95% CI, 30.0% to 100%), 75.0% (95% CI, 65.0% to 85.0%), 25.0% (95% CI, 7.7% to 42.3%), and 96.4% (95% CI, 91.6% to 100%), respectively.

**Figure 4:**
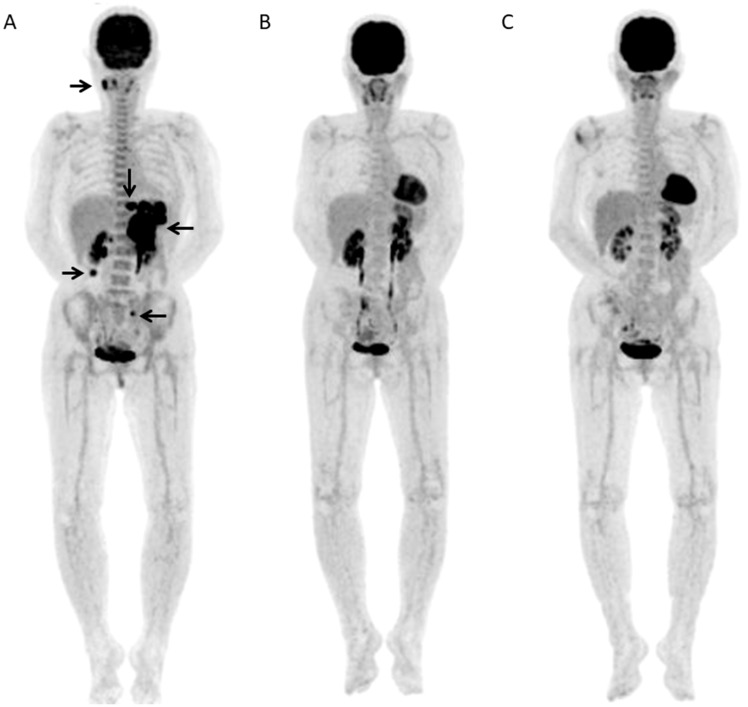
A 67-year-old female with DLBCL received 6 R-CHOP courses and no relapse was seen after 3.82 years. (**A**) Baseline FDG-PET maximum intensity projection (MIP) showed several areas of abnormal FDG uptake in right neck, abdomen, and left pelvis (arrows). (**B**) FDG-PET scan MIP after 2 courses of R-CHOP (Interim-PET) showed complete resolution of abnormal metabolic activity. (**C**) FDG-PET scan MIP after chemotherapy (End-PET) showed complete resolution of abnormal metabolic activity.

**Figure 5:**
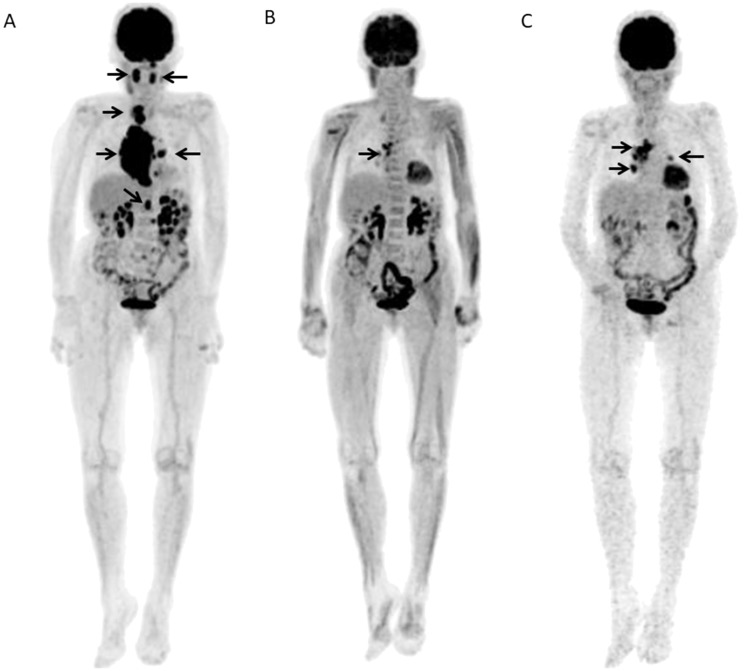
A 80-year-old female with DLBCL received 6 R-CHOP courses and then showed further progression at 0.41 years after the end of chemotherapy. (**A**) Baseline FDG-PET MIP showed several areas of abnormal FDG uptake in bilateral neck, mediastinum/hilum, and abdomen (arrows). (**B**) FDG-PET scan MIP after 3 courses of R-CHOP (Interim-PET) showed residual uptake in the mediastinum (arrows). (**C**) FDG-PET scan MIP after the chemotherapy (End-PET) showed progression of residual uptake in mediastinum and reappearance of abnormal uptake in left hilum (arrows).

Two representative cases were shown (Figures 4, 5).

## DISCUSSION

In the present series, mid-treatment FDG-PET/CT (Interim-PET after 2-4 cycles of R-CHOP therapy) results examined using visual dichotomous analysis proved to be useful for predicting PFS in patients with DLBCL receiving R-CHOP chemotherapy, though were slightly inferior to post-treatment FDG-PET/CT (End-PET) results.

Interim-PET has been reported to provide relatively good NPV and low to modest PPV findings [22]. A recent systematic review and meta-analysis demonstrated that the pooled summary false-positive proportions in patients with non-Hodgkin lymphoma were 83.0% (95% CI: 72.0%–90.2%) for Interim-PET (fixed effects, I^2^ = 27.7%) and 31.5% (95% CI: 3.9%–83.9%) for End-PET (random effects, I^2^ = 68.3%), and the authors concluded that patients with non-Hodgkin lymphoma often have a very high number of false-positive FDG-avid lesions in Interim-PET scanning findings [21]. Furthermore, Moscowitz et al. [10] analyzed Interim-PET results after 4 cycles of dose-dense R-CHOP in 97 patients with DLBCL and found that 33 of 38 found positive at the time of Interim-PET showed negativity for residual lymphoma in biopsy results.

There are some potential explanations for the high rate of false positivity. FDG does not have such a high specificity as a marker, because it is also taken up in infections and inflammatory processes, while it is also possible that use of immunotherapy may increase lesion inflammation. In addition, antibody-mediated cellular cytotoxicity and complement activation are important mechanisms related to the activity of rituximab [7, 14, 22]. Therefore, a biopsy should be considered for patients with positive PET findings if those indicate a change in treatment. On the other hand, Interim-PET is known to be associated with false-negative findings. Kwon *et al.* [17] investigated the prognostic value of negative Interim-PET results in 92 patients with DLBCL and found that 39.1% of cases shown negative later experienced relapse during the follow-up course (median 30.8 months).

Findings in the present study support the recommendation that Interim-PET should be performed only in the context of a clinical trial and is not reliable for treatment decisions in routine clinical practice. Ongoing clinical trials that alter therapy on the basis of Interim-PET may be difficult to interpret, as it is not clear that a positive FDG-PET scan result accurately defines a group of patients with poor risk for whom a change in therapy is warranted. For example, Kasamon *et al.* [8] used a risk-adapted approach to treat 59 patients with aggressive lymphoma, of whom 56 had DLBCL treated with R-CHOP. Using criteria similar to the IHP criteria, patients with a positive FDG-PET scan result after cycle 2 had their treatment changed to salvage chemotherapy, followed by autologous stem cell transplantation. More than half of their DLBCL patients had a positive interim FDG-PET scan result. For those who underwent ASCT, 74% demonstrated 2-year event-free survival (EFS), as compared to 88% of those who had a negative interim FDG-PET scan result and completed R-CHOP. Dührsen *et al.* [19] assessed whether Interim-PET guided therapy could improve outcome in 862 patients with aggressive non-Hodgkin lymphoma, including 609 with DLBCL. Those who showed R-CHOP positive results in Interim-PET after 2 cycles were assigned to receive either 6 additional cycles of R-CHOP or an intensive Burkitt protocol, whereas patients with negative FDG-PET results were assigned to either 4 additional cycles of R-CHOP, or 4 additional cycles of R-CHOP as well as 2 additional cycles of rituximab. After 2 cycles of R-CHOP, only 108 (12.5%) were classified as FDG-PET positive, whereas 754 (87.5%) were classified as FDG-PET negative. There was no significant difference regarding the EFS of patients with positive Interim-PET results and treated with 6 additional cycles of R-CHOP (2-year EFS, 42.0%) as compared with those treated with 6 additional cycles of the intensive Burkitt protocol (2-year EFS, 31.6%), and also no significant difference in EFS between Interim-PET negative patients treated with 4 additional cycles of R-CHOP (2-year EFS, 76.4%) and those treated with 4 additional cycles of R-CHOP plus 2 cycles of rituximab (2-year EFS, 73.5%). They concluded that treatment adapted based on interim FDG-PET findings did not improve outcome, but also speculated that Interim-PET could be a powerful tool to distinguish chemotherapy-sensitive from chemotherapy-resistant lymphoma.

Our study has several limitations. First, the design was retrospective and relatively few patients from a single institution were enrolled, which may have limited our ability to make generalized conclusions and introduced statistical errors. A larger prospective multicenter study is necessary to further explore and validate the potential of Interim-PET in patients with DLBCL. Second, the Interim-PET examinations were performed at different time points. Although the optimal time point remains to be established, a prospective study that utilizes the same time point of Interim-PET in the analyzed cases is needed. Third, we used the recommended standard method for evaluation of FDG-PET results, i.e., the Deauville criteria (D 5PS) [21]. On the other hand, earlier studies used custom visual criteria, such as criteria presented by the international Harmonization Project [2], the D 5PS, and maximum standardized uptake value reduction [9, 12, 15, 16, 20]. The use of different response criteria among studies makes comparisons of results challenging. We consider that consistent standardized criteria for FDG-PET/CT interpretation in this setting are needed in the future. Forth, the regimen and course of the treatment were not identical in 80 patients.

## MATERIALS AND METHODS

### Patients

This retrospective study was approved by our institutional review board, which waived the requirement for informed consent. Eighty newly diagnosed DLBCL patients referred to 3 different hematology departments between March 2009 and December 2015 were included. Demographic and tumor-related details are shown in Table 1. Each underwent FDG-PET/CT scanning at staging, then again during (Interim-PET) and at the end of (End-PET) therapy at Hyogo College of Medicine Hospital. Among all of the cases, Interim-PET was performed after 2 courses of chemotherapy for 28, after 3 courses for 34, and after 4 courses for 18 patients.

**Table 1:** Patient and tumor characteristics

Character	N	%
Sex		
Male	45	56.3
Female	35	43.7
Age		
Mean	64.3 ± 13.6	
Range	22–84	
Lactate dehydrogenase		
Normal	35	43.8
Abnormal	45	56.3
Initial clinical stage		
I	15	18.8
II	23	28.7
III	12	15
IV	30	37.5
IPI risk group		
Low	28	35.0
Low-intermediate	22	27.5
High-intermediate	16	20.0
High	14	17.5
Treatment		
R-CHOP	61	76.3
R-THP-COP	19	23.7
Total courses of chemotherapy		
Six courses	56	70.0
Eight courses	24	30.0
Timing of Interim-PET after chemotherapy		
After two courses	28	35.0
After three courses	34	42.5
After four courses	18	22.5

Abbreviaitons: IPI: International prognostic index; R-CHOP: rituximab, plus cyclophosphamide, doxorubicin, vincristine, and prednisone; R-THP-COP: rituximab, pirarubicin, cyclophosphamide, vincristine, and prednisone.

All treatments were performed according to departmental protocol. Patients were given standard R-CHOP (*n =* 61) or R-THP-COP (rituximab, pirarubicin, cyclophosphamide, vincristine, prednisone) (*n =* 19) for 6 (*n =* 56) or 8 (*n =* 24) courses. Therapy for each was performed as planned and never modified based on Interim-PET results. Involved field radiotherapy was performed to areas of bulky disease regardless of PET results (*n =* 8).

### PET scanning

PET scans (Gemini GXL16 or Gemini TF64; Philips Medical Systems, Eindhoven, The Netherlands) were performed for all patients at the time of diagnosis, during (Interim-PET) after 2, 3, or 4 cycles of chemotherapy, and at the end (End-PET) of treatment at Hyogo College of Medicine Hospital. Patients were instructed to fast for 5 hours before the scan, and blood glucose was measured immediately before injection of FDG at 4.0 MBq/kg body weight for the GXL16 or 3.0 MBq/kg for the TF64. None of the present cohort had a blood glucose level greater than 150 mg/dL. Static emission images were obtained approximately 60 minutes after injection. For attenuation correction and anatomic localization, helical CT scans from the top of the head to the bottom of the feet were obtained using the following parameters: tube voltage, 120 kV; effective tube current auto-mA (up to 120 mAs for GXL16, up to 100 mAs for TF64); gantry rotation speed, 0.5 seconds; detector configuration, 16 × 1.5 mm for GXL16, or 64 × 0.625 mm for TF64); slice thickness, 2 mm; and transverse FOV, 600 mm. Immediately after completion of CT scanning, PET images of the region from the head to mid-thigh were acquired for 90 seconds per bed position and from the mid-thigh to toes for 30 seconds per bed position employing the variable sampling method. Thereafter, images at 12–14 bed positions, each for 90 seconds, and 6–7 bed positions, each for 30 seconds, were acquired in three-dimensional mode, thus requiring between 22 to 26 minutes of emission scanning per patient. The patients were allowed to breathe normally during PET acquisitions. Attenuation-corrected PET images were reconstructed using a line-of-response row-action maximum likelihood algorithm (n/a subsets, 2 iterations) for the GXL16 or an ordered-subset expectation maximization iterative reconstruction algorithm (33 subsets, 3 iterations) for the TF64.

### PET findings interpretation

Baseline, Interim-PET, and End-PET scan images were retrospectively reviewed by 2 experienced nuclear medicine physicians (10 and 2 years of experience with oncologic FDG-PET/CT scanning, respectively) who had no knowledge of the other imaging results, or clinical and histopathologic results other than those obtained before, during, and after chemotherapy for DLBCL, with consensus obtained by discussion.

All Interim-PET and End-PET results were interpreted as positive or negative by visual dichotomous response criteria using a 5-point score system defined as the Deauville criteria (D 5PS) [22], as follows: 1, no uptake; 2, uptake equal or less than mediastinum; 3, uptake more than mediastinum but less than liver; 4, uptake moderately increased as compared with liver at any site; 5, uptake markedly increased as compared with liver at any site and/or new site of disease. A score of 1 through 3 was regarded as indicating negative and that of 4 or 5 as indicating positive findings.

### Statistical analysis

PFS was chosen as the study endpoint, and was defined as the time from diagnosis to either disease progression or relapse, or to death from any cause. Data were censored if the patient was alive and free of progression/relapse at the final follow-up examination. Survival curves were calculated using the method of Kaplan and Meier according to the Interim-PET or End-PET result (positive *vs.* negative). Log-rank tests were performed, with p-values obtained to estimate survival probability after 2 years.

For predicting relapse or disease progression, sensitivity, specificity, positive predictive value (PPV), and negative predictive value (NPV) with the 95% confidence interval (95% CI) were analyzed for both Interim-PET/CT and End-PET/CT scanning results.

Each test was 2-sided and the level of significance was set at 0.05. All analyses were performed using SAS software.

### Ethical approval

All included patients gave written and informed consent for the diagnostic procedure. Due to the retrospective character of the patient data evaluation ethical commitment was waived by the institutional ethics board.

## CONCLUSIONS

In conclusion, mid-treatment FDG-PET/CT may prove useful for providing predictive information for patients with DLBCL undergoing R-CHOP chemotherapy. Nevertheless, a larger prospective multicenter study is necessary to further explore and validate the potential of Interim-PET in patients with DLBCL. Furthermore, mid-treatment FDG-PET/CT results should not be used at this time in routine clinical practice to make treatment decisions.

## Abbreviations

FDG: ^18^F-fluorodeoxyglucose
PET/CT: positron emission tomography/computed tomography
R-CHOP: rituximab, plus cyclophosphamide, doxorubicin, vincristine, and prednisone
DLBCL: diffuse large B-cell lymphoma
PFS: progression-free survival
PPV: positive predictive value
NPV: negative predictive value
ASCT: autologous stem cell transplantation
R-THP-COP: rituximab, pirarubicin, cyclophosphamide, vincristine, prednisone
95% CI: 95% confidence interval
EFS: event-free survival
